# A synthetic standard for the analysis of carbon isotopes of carbon in silicates, and the observation of a significant water-associated matrix effect

**DOI:** 10.1186/s12932-015-0029-x

**Published:** 2015-09-15

**Authors:** Christopher H. House

**Affiliations:** Department of Geosciences, Penn State Astrobiology Research Center, 220 Deike Building, University Park, PA 16802 USA

**Keywords:** Carbon isotopes, SIMS, Standardization, Matrix effect

## Abstract

**Background:**

Due to the biogeochemical fractionation of isotopes, organic material can be heterogeneous at the microscale. Because this heterogentiy preserves in the rock record, the microscale measurement of carbon isotopes is an important frontier of geobiology. Such analyses via secondary ion mass spectrometry (SIMS) have been, however, held back by the lack of an appropriate homogeneous synthetic standard that can be shared between laboratories. Such a standard would need to yield a carbon signal intensity within the same instrument dynamic range as that found for typical rocks, exhibit minimal matrix effects under typical SIMS conditions, and be widely available. In this work, five possible standards were tested with repeated δ^13^C ion microprobe measurements against the PPRG #215-1 Precambrian chert that has been used as a working standard for these types of analyses by several laboratories.

**Results:**

Results showed that silica powder, pressed, and bonded with Ceramacast 905 produced a useful synthetic standard. The material produced has a secondary ion carbon yield of only about 15× that of the PPRG #215-1 organic-rich chert. Finally, the material, once dried sufficiently, did not demonstrate an observable matrix effect when the carbon isotopes were measured. Another similar material (silica nanopowder, pressed, and bonded with Aremco-Bond 526N) appears to have retained its hydration after a substantial effect to dry it. The isotopes measurements of this more hydrated material showed a significant matrix effect that was diminished by intense pre-sputtering. The results indicate water can affect SIMS carbon isotopic measurements, and an intense beam reduces the effect. A hydrated standard might be useful to monitor the effect.

**Conclusions:**

A suitable artificial standard for SIMS isotopic measurements of organic material in rocks has been found, and it will allow an acute growth in both the quantity and quality of studies of ancient carbon at the microscale. Also, this work has revealed a novel water-associated matrix effect for carbon isotopes. This newly revealed matrix effect is important because it might have misled previous research. The effect could lead to increased observed heterogeneity of partially hydrated samples and/or produced systematic differences between natural targets and the standards used.

## Background

Efforts to understand the long Precambrian history of life on Earth have generally focused on either the morphology of microfossils preserved, the geochemical signatures of specific metabolisms, or the preservation of organic material in sedimentary rocks. In particular, the carbon isotopic analyses of bulk organic material extracted from rocks has been particularly important for showing the antiquity of life on Earth and major perturbations of the global carbon cycle. Interpretations of bulk carbon isotope measurements are, however, limited because they represent an average of carbon in rocks, which is a mix of carbon from indigenous organisms, reworked organic material, and potentially post-depositional contaminants. Microanalysis of carbon isotopes allows researchers to obtain carbon isotopic information for individual microfossils, as well as reveal diversity of microbi al metabolisms in an environment from carbon isotopic microscale heterogeneity. The work published to date demonstrates that there is a great deal of such heterogeneity that is masked in a bulk analysis (e.g., [1–7]). This heterogeneity could, if measured, be a signature of particular microbial processes occurring when the sediments were deposited. For example, different carbon fixation pathways impart demonstrated different carbon isotopic fractionations [8–10], and different sources of carbon could result in sedimentary organic material with distinct carbon isotopic compositions (e.g., [11–13]).

In spite of the high value carbon isotopic microanalyses have for the fields of biogeochemistry and geobiology, the number of isotope studies of ancient sedimentary carbon conducted at the microscale remains quite small. The primary barrier to a substantial increase in work on this topic has been the lack of an appropriate homogeneous synthetic standard that can be shared between laboratories worldwide. In SIMS carbon isotope research, a standard of known isotopic composition is needed that can be analyzed in parallel with the materials being studied. The lack of an ideal standard has both hindered experienced secondary ion mass spectrometry (SIMS) laboratories and new potential researchers to the field.

For past work, several studies have used powdered graphite to study natural graphite or organic material in silicate rocks [14–16]. Under at least some SIMS conditions, however, there appears to be a matrix effect between graphite and organic material [3, 5], and there are reports that H/C can correlates with a matrix effect [17, 18]. Diamond has also been used as a standard for SIMS work [19], and it remains a viable option for these types of measurements. The possible limitations with diamond are that there might be a matrix effect between diamond and sedimentary organic material, and that diamond is hard to share between disparate laboratories. Also, while not well documented in the literature, several groups have tried using either epoxy or plastics as a carbon standard for organic material. This approach could be useful as an isotope standard for analyses of very organic-rich materials, but the approach is problematic for analyses of typical sedimentary rocks that contain only a few percent carbon. The problem is that the carbon-rich standard gives a much stronger signal than the unknowns and therefore must be analyzed under different conditions and potentially with a different detector set-up. Several groups have used the Precambrian rock chip PPRG #215-1 as a working standard for the analysis of carbon isotopes of organic material in Precambrian cherts [3, 7, 20, 21]. The advantage of this approach has been the lack of any significant matrix effect between the standard and other natural unknown samples. The downsides have been that it is hard to share between disparate laboratories, and that there is some isotopic heterogeneity making it hard to use for the evaluation of ion microprobe spot-to-spot reproducibility. Also, the carbon secondary ion signal obtained from PPRG #215-1 is often less than that obtained from large microfossils with the same SIMS conditions.

The ideal standard for isotope measurements via SIMS would yield a secondary carbon signal intensity within the same instrument dynamic range as that typically-found for microfossils in sedimentary rocks, exhibit minimal matrix effects under typical SIMS conditions used, and be available to the whole geochemical community. Here, we have investigated five possible carbon SIMS standards. The candidate materials were either synthesized, purchased, or donated and were tested with repeated δ^13^C ion microprobe measurements against the PPRG#215-1 Precambrian rock chip. We report, here, that one of these materials tested is quite suitable for SIMS work. During the testing, I discovered a matrix effect associated with the degree of hydration of the material being analyzed, and I found that an intense pre-sputter reduces the observed effect.

## Results and discussion

### Secondary ion yields

Table 1 shows the yield of carbon secondary ions (in this case C_2_^−^) for each of the five materials studied here along with the yield for PPRG #215-1. This initial test demonstrated that two of the five materials (Ceramacast 905 + silica and Aremco-Bond 526N + silica) produce secondary ion yields in a similar magnitude to typical natural organic-rich sedimentary rocks. From these measurements, it appears that one of these materials could be a useful isotope standard for natural samples. The other three materials produced secondary ion yields that were a few orders of magnitude higher. From these results, it is quite possible that these other materials could be useful standards when analyzing very carbon-rich materials. In particular, because PEEK GF30 is a commercially available high performance glass-filled plastic, its overall elemental composition is similar to natural samples and it is readily available. Thus, PEEK GF30 (with or without added surface silica) would likely be a useful standard for analyses of coal or large particulate organic material extracted from rocks (including, for example, extracted Acritarchs). Also, with more silica nanopowder adhered to the surface, PEEK might be an excellent standard for the SIMS analyses of a variety of targets. However, additional work is needed on a procedure to produce such a material.

**Table 1:** Observed carbon ion yields for different materials

Possible standard material	Approx. C_2_ yield (10^6^ cps per nA)
PPRG #215-1	0.1^a^
PEEK GF30 + silica	75
Ceramacast 905 + silica	1.5
Aremco-Bond 526N + silica	1.5
ADVANCER CN703 (bonded SiC)	35
CARBOFRAX A (bonded SiC)	50

^a^Signal varies between different spots by about an order of magnitude on this natural rock chip

### Carbon isotopic measurements

**Fig. 1:**
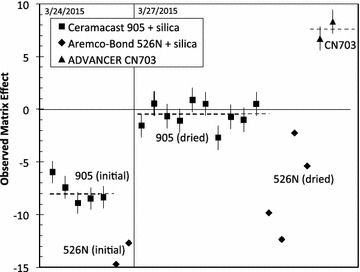
Observed matrix effect during ion microprobe carbon isotopic analyses between three synthetic materials and the PPRG #215-1 Precambrian chert that has been used as a working standard previously

In order to investigate the utility of the three materials with carbon signals most similar to that of natural sedimentary silicates (i.e., Ceramacast 905 + silica, Aremco-Bond 526N + silica, and ADVANCER CN703), we used an ion microprobe to measure their carbon isotopic composition using the PPRG #215-1 as a standard. By using a natural rock standard, this test was a measure of observable matrix effects during the isotope measurement by SIMS. In general, SIMS is known for potentially having large instrumental mass fractionations that need to be corrected by a parallel analysis of a standard. Figure 1 shows the observed matrix effects of each material on two different days. The first day of analyses (March 24th) was shortly after synthesizing, mounting, and polishing the materials. The second set of analyses was conducted 3 days later. Between these two ion microprobe sessions, the materials were stored in a heated vacuum oven.

The δ^13^C results from March 24th clearly show a large matrix effect with the 905 and 526N samples appearing more depleted in ^13^C than they are. The matrix effect observed for the 526N sample was larger than that of 905 at up to 14‰. Because I had observed swelling of the 526N sample during polishing and rinsing (see experimental methods), I hypothesized that the matrix effect could be associated with hydration.

**Fig. 2:**
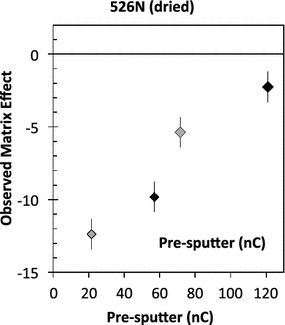
Observed matrix effect during ion microprobe carbon isotopic analyses between a hydrated material (the 526N sample) and the PPRG #215-1 Precambrian chert shown as a function of the total intensity of the pre-sputter. The* larger diamonds* had an additional 20 s pre-sputter using a 2 nA beam.* Grey diamonds* using an analysis primary beam of 0.4 nA, while the* black diamonds* are cases were the analysis primary ion beam was ~1 nA. The results suggest the stronger additional pre-sputter (at 2 nA) and a more intense analysis primary ion beam (at 1 nA) both reduce the observed matrix effect

Based on the hypothesis that at least part of the matrix effect observed was associated with water, the samples were reanalyzed after being in the vacuum oven. The δ^13^C results from March 27th provide support for the notion that water is associated with a substantial matrix effect. The later results from Ceramacast 905 + silica showed no significant matrix effects and were constant over the course of each run. In contrast, the more hydrated Aremco-Bond 526N + silica again showed a large matrix effect (with the carbon appearing to be up to 12‰ more depleted in ^13^C than it is). Because a more intense primary ion beam appeared to reduce the observed effect (Fig. 1), I added an intense pre-sputtering using a ~3 nA beam before the final two δ^13^C measurements of the 526N sample. The March 27th results from the 526N sample show that both an increased primary ion beam and the addition of an intense pre-sputter greatly reduce the observed matrix effect (Fig. 2). These results suggest that the effect is due to hydration that occurred during sample preparation. The synthetic standards, generally, did not show significant trends toward more ^13^C-depleted values over the course of individual analysis runs, as might be expected if the pre-sputter was simply removing the hydrated surface. Overall, the results still indicate that sample drying, adequate intense pre-sputtering, and an intense primary ion beam reduce matrix effects between the different samples.

At this time, it is not clear how water generates such a large matrix effect. We can, however, rule out some possible mechanistic options. First, this is not due to interference from the molecular hydride ion, as that would result in the appearance of ^13^C-enrichment rather than ^13^C-depletion. Likewise, this is not exclusively a sample chamber vacuum issue, because the effect was observed between two materials (PPRG #215-1 and Ceramacast 905) mounted on the same grain mount.

The effect observed here does operate in the same direction as the matrix effect correlated with H/C observed by [17, 18] where higher hydrogen was correlated with spots appearing to be more ^13^C-depleted. These various results might be related if these matrix effects result from increased ionization of ^12^C and C_2_, which could occur in the presence of both water and hydrogen. It should be noted, though, that the organic materials used in this present study are quite rich in hydrogen and so it does not appear that H/C was associated with a matrix effect for the SIMS conditions used here. Also, based on the observation that a 3 nA pre-sputter was effective at reducing the matrix effect observed here, we might not expect this effect under the conditions used by [17] which had primary ion beams of between 2 and 20 nA.

It is also unclear why an intense pre-sputter lessens or eliminates the matrix effect observed here. If the matrix effect is related to ionization efficiency, the cesium added during the intense pre-sputter might moderate the influence of the water present. This explanation, however, seems erroneous because the intense pre-sputter is resulting in a material that acts like the water-poor PPRG 215-1 even though cesium increases overall ionization. Another possible explanation is that some hydration slightly increases the electrolytic conductivity of the surface. In this explanation, an intense pre-sputter could reduce the observed matrix effect by a combination of removing surface layers that are particularly wet and dehydrating a zone beneath the pit through ion milling. Regardless of the cause of the matrix effect, it might be possible to incorporate the Aremco-Bond 526N + silica material into a working test of SIMS conditions prior to the analysis of important samples to ensure that the conditions used are suitable for minimizing water-associated matrix effects. For the Ceramacast 905 plus silica material, 60 s of pre-sputter was used on March 27th, which was sufficient for the results reported, and so it is unclear if a more intense pre-sputter of the Ceramacast 905 would have removed the about 0.5‰ difference in instrumental mass fractionation seen in the reported data (Fig. 1).

Last, the ADVANCER CN703 sample was analyzed. Although this silicon carbide material had demonstrated a high carbon secondary ion signal, its carbon signal could be analyzed using the same ion microprobe configuration by greatly reducing the intensity of the primary ion beam. The results shown in Fig. 1 demonstrate a significant matrix effect with the material appearing more enriched in ^13^C than it actual composition by almost 6‰. At face value, these results indicate that like graphite, SiC is not a proper SIMS standard for organic carbon.

## Experimental

I chose five materials to be tested for their suitability as isotope standards for δ^13^C analyses of carbon in silicate sedimentary rocks. These materials either were purchased, donated, or synthesized for this research. The materials were then analyzed for their δ^13^C composition using the UCLA CAMECA ims 1270 ion microprobe.

### PEEK GF30 + silica synthesis

A non-annealed, one inch-diameter rod of PEEK GF30 was donated to the project by Quantum Polymers. PEEK was selected because it is a high performance thermoplastic reducing the possibility that it would soften or ablate during the sputtering process or if mistakenly hit with high-energy electrons. A glass-filled variety was selected to have SiO_2_ be a major component of the material, as is the case for sedimentary silicates. During previous research, we had found that PEEK was isotopically homogeneous, but generates too strong a secondary ion beam for comparison with Proterozoic cherts (Peng, Guo, House, Chen, and Ta, unpublished observations). In this work, I attempted to reduce the secondary ion signal from PEEK to a level similar to that of natural sedimentary silicates by adding a mask of silica to the top of the GF30 disk. To do this, in short, I placed a PEEK disk into silica powder and then heated the disk beyond the melting point of PEEK so that some of the silica would adhere to the plastic surface.

**Fig. 3:**
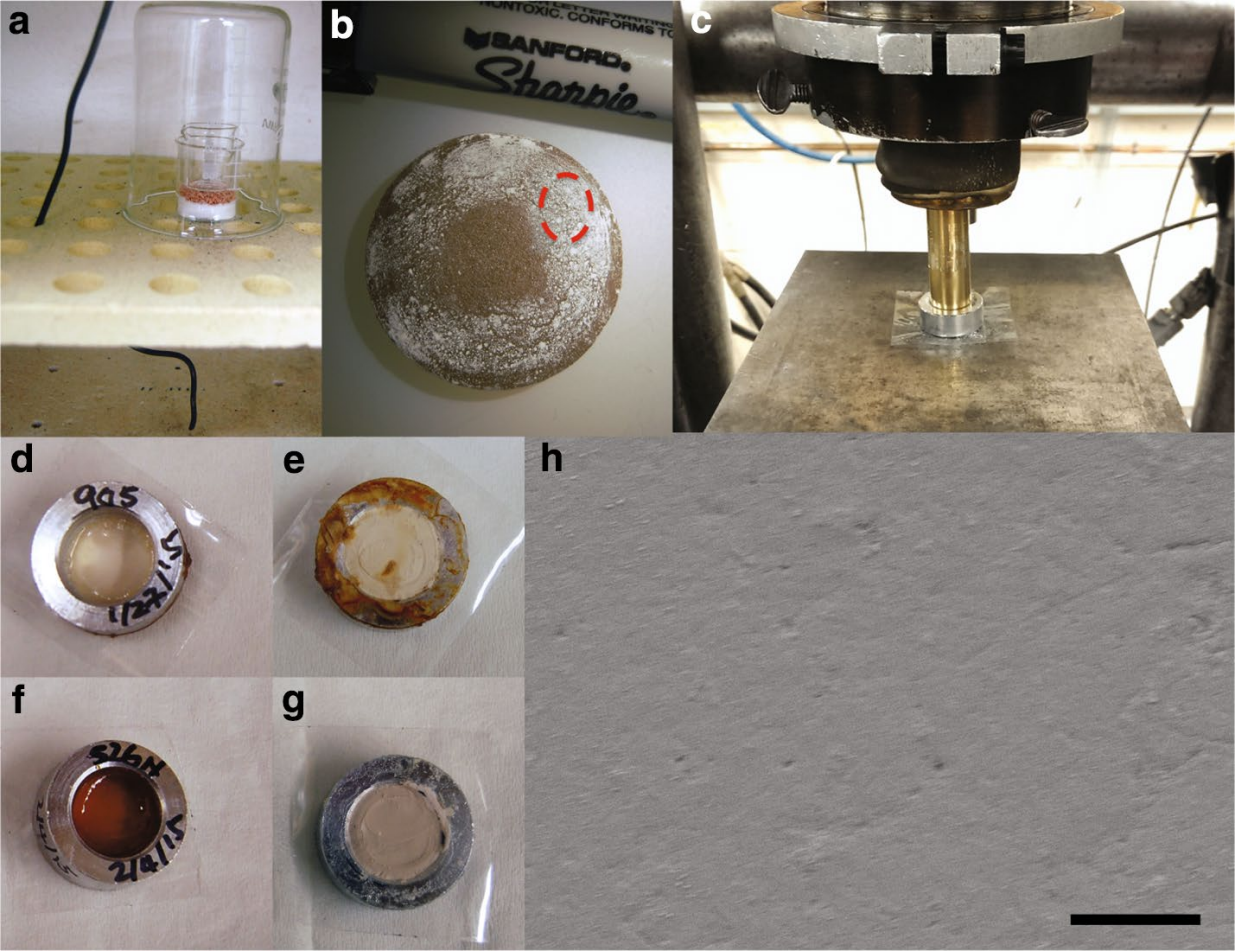
Images showing the syntheses of three of the materials used in this study. **a** A PEEK GF30 disk surrounded by silica nanopowder and covered by a layer of copper filings, in an oven to be heated to 345 °C under a nitrogen flow. **b** PEEK GF30 disk after removal from the oven showing silica nanopowder adhered to the surface. The red circle shows the approximate piece cut out and used for the test. **c** Silica nanopower in an aluminum ring being pressed at 25 MPa. **d** Pressed silica nanopowder with Ceramacast 905 added. **e** Same sample as D turned over to show side that will be polished after the packing tape is removed. **f** Pressed silica nanopowder with Aremco-Bond 526N added. **g** Same sample as F turned over to show side that will be polished after the packing tape is removed. **h** SEM image of the surface of the polished Ceramacast 905 + silica sample used for SIMS.* Scale bar* is 20 μm

First, a ~0.5 cm long piece of the PEEK GF20 rod was cut forming a disk. A bed of 0.5 g of silica nanopowder (Sigma-Aldrich item #637238) was placed into a 25-mL Pyrex beaker. The PEEK disk was then placed onto this bed of silica powder, and another 0.5 g of silica nanopowder was placed on top of the disk, covering it completely. A 15-mL beaker was then placed on top of the silica, and approximately one gram of copper filings was added to that beaker to act as an oxygen scrub later. The whole set-up was then placed into an ashing furnace covered by a large beaker (Fig. 3a). A copper line was run into the furnace to continuously flow nitrogen though the oven space. The oven temperature was then raised to 345 °C over the course of a couple of hours. Then, the oven temperature was held at 345 °C for 1 h. The melting point of PEEK is 343 °C and so this procedure was meant to ensure that the PEEK disk exceeded its melting point while surrounded by silica powder that could then adhere to the plastic (Fig. 3b). This procedure was not optimized, and certainly could use improvement if this material is to become a widely used standard. For example, placing the sample between two metal blocks would likely provide more even heating and pressure (Katherine Freeman, personal communication).

### Ceramacast 905 + silica synthesis

Ceramacast 905 is a one component high temperature casting silicone rubber (purchased from Aremco). This casting material was used to bond the grains of a pressed silica powder producing a solid disk of mostly silica and secondarily rubber.

First, a 1-inch OD aluminum ring (“aluminum unthreaded spacer,” item #92510A818 from McMaster-Carr) was taped on one side with acrylic packing tape. 0.5 g of silica nanopowder (Sigma-Aldrich item #637238) was then pressed into the ring at ~25 Mpa by a brass piston using a biaxial loading frame (Fig. 3c). Then, 0.5 g of the Ceramacast 905 liquid reagent was added to the ring (Fig. 3d). The ring was set aside for 24 h to air dry (allowing the volatile component of the reagent to leave). The ring was then heated for 2 h at 230 °C to cure the rubber. This one-step curing approach was used rather than the manufacturer’s directions because it appeared that by going to 230 °C immediately the rubber migrated throughout the silica before hardening. After curing, the tape was carefully removed from the underside of the sample (Fig. 3e), and the exposed surface was then polished by hand using a slurry of 3-micron alumina (Buehler) on a polishing cloth adhered to a glass plate. The polished material was later removed from the aluminum ring and placed in an epoxy grain mount.

Figure 3h shows a scanning electron microscope (SEM) image of the polished surface of the Ceramacast 905 + silica sample. The SEM image shows that the silica grains are small relative to a typical dynamic SIMS primary ion beam. However, the surface, as prepared here, may not be suitable for NanoSIMS without additional refinement becuase NanoSIMS is often operated with sub-micrometer-sized primary ion beams.

### Aremco-Bond 526N + silica synthesis

Aremco-Bond 526N is a two component high temperature epoxy (purchased from Aremco). This epoxy was used to bond the grains of a pressed silica powder producing a solid disk of mostly silica and secondarily rubber.

First, another 1-inch OD aluminum ring (item #92510A818 from McMaster-Carr) was taped on one side with acrylic packing tape. 0.5 g of silica nanopowder (Sigma-Aldrich item #637238) was then pressed into the ring at ~25 Mpa by a brass piston using a biaxial loading frame. Then, 0.6 g of the Aremco-Bond 526N epoxy mixture was added to the ring (Fig. 3f). The ring was then heated at 170 °C for 3.5 h to cure the epoxy. After curing, the tape was carefully removed from the underside of the sample (Fig. 3g), and the exposed surface was then polished by hand using a slurry of 3-micron alumina on a polishing cloth adhered to a glass plate. In this case, the aluminum ring was cut in half to decrease the depth of the sample and allow the whole mount to fit into a typical SIMS sample holder.

During the polishing step, the sample swelled (as the silica apparently hydrated), and then popped out of the aluminum ring due to its swelling. In contrast, this clear swelling was not observed for the 905 sample when it was similarly polished.

### ADVANCER CN703 and CARBOFRAX a procurement

ADVANCER CN703 and CARBOFRAX A ceramic plates were donated by Saint-Gobain Materials. 1-cm size squares were cut from this silicon carbide-based material. The cut-surfaces were used for the ion microprobe analyses after being placed in an epoxy grain mount.

## Conclusions

This study has shown that silica powder, pressed, and then bonded with Ceramacast 905 produced a useful synthetic standard. The material produced has a secondary ion carbon yield of only about 15× that of the organic-rich chert PPRG #215-1 and similar to large microfossils in polished chert sections. Finally, the material, once dried sufficiently, did not demonstrate a significant matrix effect when the carbon isotopes were determined. The resultant suitable carbon isotope standard for SIMS measurements of organic material in rocks will allow an acute growth in both the quantity and quality of studies of ancient carbon at the microscale.

The project has revealed a strong matrix effect associated with hydration of two of the materials studied. Especially, silica powder, pressed, and then bonded with Aremco-Bond 526N clearly showed this water-associated matrix effect, and repeated analyses of the 526N sample demonstrated that the effect was greatly reduced by an intense pre-sputter. This newly revealed matrix effect is important because it might have misled previous research. The effect could lead to increased observed heterogeneity of partially hydrated samples and/or produced systematic differences between natural targets and the standards used.

## Methods

### Ion microprobe analyses

δ^13^C compositions were determined with the University of California–Los Angeles (UCLA) Cameca 1270 using a multicollector configuration with ^12^C_2_^−^ detected by an off-axis electron multiplier (EM) and ^12^C^13^C^−^ measured using the central EM. The molecular ion (C_2_^−^) produces a stronger count rate than the atomic ion (C^−^), and its mass permits the on-axis detector to be used. SIMS was performed using a 0.03–1.2 nA, ~8 μm, Cs^+^ primary beam (with lower intensities required for ADVANCER CN703 and a range of intensities used for the PPRG #215-1). The mass resolving power (M/M) was about 6000, and charge compensation was achieved using a normal incident electron gun and a gold coat. The analyses (Table 2) were calibrated against repeated analyses of PPRG#215–1 Precambrian chert, as previously used for Precambrian microfossils [3, 7, 20, 21]. The instrumental mass fractionation was found to be similar to past experience with these conditions. Under these conditions, quasi-simultaneous arrival effects are expected to be small because the production of C_2_^−^ is relatively ineffective with ionization around a few percent. In most cases, a pre-sputter of the analysis primary ion beam was used. These conventional pre-sputter times are listed in Table 2. For the final four analyses of Aremco-Bond 526N + silica, pre-sputter was added with combinations of 60 s of a 0.4 nA primary ion beam and/or 20 s of a ~3 nA primary ion beam.

**Table 2:** Carbon isotope analyses by ion microprobe of possible synthetic standards

Sample material	Primary Cs^+^ intensity	Pre sputter (s)	C_2_^−^ intensity (cps)	Observed δ^13^C ± σ	PPRG #215-1 Instrument mass fractionation factor α	Corrected δ^13^C	True δ^13^C	Observed matrix effect
905	6.7E−10	15	1.7E+05	−33.1 ± 1.1	1.0006 (0.6 ‰)	−33.7	−27.9	−6.0
905	6.7E−10	15	3.4E+05	−34.5 ± 0.7	1.0006 (0.6 ‰)	−35.1	−27.9	−7.4
905	2.2E−10	15	3.7E+05	−35.9 ± 0.7	1.0006 (0.6 ‰)	−36.5	−27.9	−8.9
905	2.2E−10	45	2.6E+05	−35.5 ± 0.7	1.0006 (0.6 ‰)	−36.1	−27.9	−8.4
905	2.2E−10	45	2.3E+05	−35.4 ± 1.1	1.0006 (0.6 ‰)	−36.0	−27.9	−8.4
					Weighted mean	*−35.7*		*−8.0*
					Weighted standard deviation	*1.0*		
526N	8.30E−11	45	1.3E+05	−41.9 ± 0.7	1.0006 (0.6 ‰)	−41.9	−27.7	−14.7
526N	8.40E−11	45	7.6E+05	−40.0 ± 1.1	1.0006 (0.6 ‰)	−40.0	−27.7	−12.7
					Weighted mean	*−40.4*		*−13.1*
					Weighted standard deviation	*1.4*		

Sample material	Primary Cs^+^ intensity	PrE−sputter (s)	C_2_^−^ intensity (cps)	Observed δ^13^C ± σ	PPRG #215-1 Instrument mass fractionation factor α	Corrected δ^13^C	True δ^13^C	Observed matrix effect
905	7.5E−11	60	1.2E+05	−24.9 ± 1.4	1.0046 (4.6 ‰)	−29.4	−27.9	−1.6
905	1.1E−10	60	2.6E+05	−22.9 ± 0.8	1.0046 (4.6 ‰)	−27.4	−27.9	0.5
905	5E−11	60	1.4E+05	−24.0 ± 1.2	1.0046 (4.6 ‰)	−28.5	−27.9	−0.7
905	5.2E−11	60	1.6E+05	−24.4 ± 1.0	1.0046 (4.6 ‰)	−29.0	−27.9	−1.1
905	4.1E−11	60	1.4E+05	−22.5 ± 1.4	1.0046 (4.6 ‰)	−27.0	−27.9	0.9
905	5E−11	60	1.6E+05	−22.9 ± 0.8	1.0046 (4.6 ‰)	−27.4	−27.9	0.5
905	1E−10	60	2.1 E+05	−26.0 ± 0.8	1.0046 (4.6 ‰)	−30.5	−27.9	−2.7
905	1E−10	60	2.0E+05	−24.1 ± 1.1	1.0046 (4.6 ‰)	−28.6	−27.9	−0.7
905	1.6E−10	60	2.4E+05	−24.4 ± 1.1	1.0046 (4.6 ‰)	−28.9	−27.9	−1.0
905	1.6E−10	60	3.0E+05	−22.9 ± 0.8	1.0046 (4.6 ‰)	−27.4	−27.9	0.5
					Weighted mean	*−28.4*		*−0.5*
					Weighted standard deviation	*1.1*		
526N	9.5E−10	60	1.2E+05	−32.8 ± 2.0	1.0046 (4.6 ‰)	−37.2	−27.7	−9.8
526N	3.6E−10	60	3.9E+04	−35.2 ± 3.5	1.0046 (4.6 ‰)	−39.7	−27.7	−12.4
526N	9.4E−10	60+	2.7E+05	−25.4 ± 1.2	1.0046 (4.6 ‰)	−29.9	−27.7	−2.3
526N	3.6E−10	60+	6.2E+04	−28.4 ± 2.1	1.0046 (4.6 ‰)	−32.9	−27.7	−5.4
					Weighted mean	*−32.5*		*−4.9*
					Weighted standard deviation	*4.5*		
CN703	3.1E−11	60	4.5E+05	−17.5 ± 1.1	1.0046 (4.6 ‰)	−22.1	0.0	6.7
CN703	3.1E−11	60	2.5E+05	−15.9 ± 1.2	1.0046 (4.6 ‰)	−20.5	0.0	8.3
					Weighted mean	*−21.3*		*7.4*
					Weighted standard deviation	*1.1*		

Instrument mass factionation (IMF) factor α = (δ^13^C_obs_ + 1000)/(δ^13^C_Exp_ + 1000)

IMF in ‰ = 1000 * Ln (α). Corrected δ^13^C = ((δ^13^C_obs_ + 1000/α) − 1000

True δ^13^C is that measured by conventional elemental analyzer-isotope ratio mass spectrometry (EA-IRMS). apparent matrix effect (AS) = Corrected δ^13^C − True δ^13^C

The last two 526N samples add the following additional pre-sputter respectively: 20 seconds of an ~2 nA beam and 60 s of an ~0.4 nA beam; 20 s of an ~2 nA beam

### Scanning electron microscopy

An SEM image at 2500× magnification was taken of the polished and gold-coated surface of the Ceramacast 905 + silica sample. The SEM high voltage was 20 kV, the working distance was 10 mm, the spot diameter was 5 nm, and the horizontal field width was 0.11 mm.

## Abbreviations

SIMS: secondary ion mass spectrometry is a form of mass spectrometry where a focused primary ion beam is used to sputter the surface of a specimen ejecting secondary ions. The secondary ion beam generated is then analyzed by measuring the mass-to-charge ratio
PEEK: polyether ether ketone is a colorless high-performance thermoplastic polymer
SEM: scanning electron microscope is a microscope that forms a image by scanning a target with a focused beam of electrons
